# Supplementary material for: USP7 deubiquitinase stabilizes FAN1 to support DNA crosslink repair and suppress CAG repeat expansion
Source: Nat Commun. 2026 Mar 6;17:3551. doi: 10.1038/s41467-026-70051-9 (PMC13086964; doi:10.1038/s41467-026-70051-9)
Supplement: Supplementary file 1 — Supplementary Information [file 41467_2026_70051_MOESM1_ESM.pdf]

Supporting Information

USP7 deubiquitinase stabilizes FAN1 to support DNA crosslink repair and suppress CAG repeat expansion

Giulio Collotta[1], Marco Gatti[1], Irina-Maria Ungureanu[2], Vanessa van Ackeren[1], Emilie Rannou[3], Francesca Vivalda[1], Diego Gomez Vieito[1], Keri M Fishwick[1], Christine von Aesch[1], Antonio Porro[1], Kyra Ungerleider[2], Ailin Heidari[4,5,6], Raphaël Guérois[7], Rachel J Harding[4,5,6], Sylvain Bischof[3], Gabriel Balmus[2,8,*], Alessandro A Sartori[1,*]

[1]Institute of Molecular Cancer Research, University of Zurich, Zurich, Switzerland.

[2]UK Dementia Research Institute, Department of Clinical Neurosciences, University of Cambridge, Cambridge Biomedical Campus, Cambridge, UK.

[3]Department of Plant and Microbial Biology, University of Zurich, Zurich, Switzerland.

[4]Structural Genomics Consortium, University of Toronto, Toronto, Ontario, Canada.

[5]Department of Pharmacology & Toxicology, University of Toronto, Toronto, Ontario, Canada.

[6]Leslie Dan Faculty of Pharmacy, University of Toronto, Toronto, Ontario, Canada.

[7]Université Paris-Saclay, CEA, CNRS, Institute for Integrative Biology of the Cell (I2BC), 91198, Gif-sur-Yvette, France.

[8]Department of Molecular Neuroscience, Transylvanian Institute of Neuroscience, Cluj-Napoca, Romania.

[*]e-mail: gb318@cam.ac.uk; sartori@imcr.uzh.ch

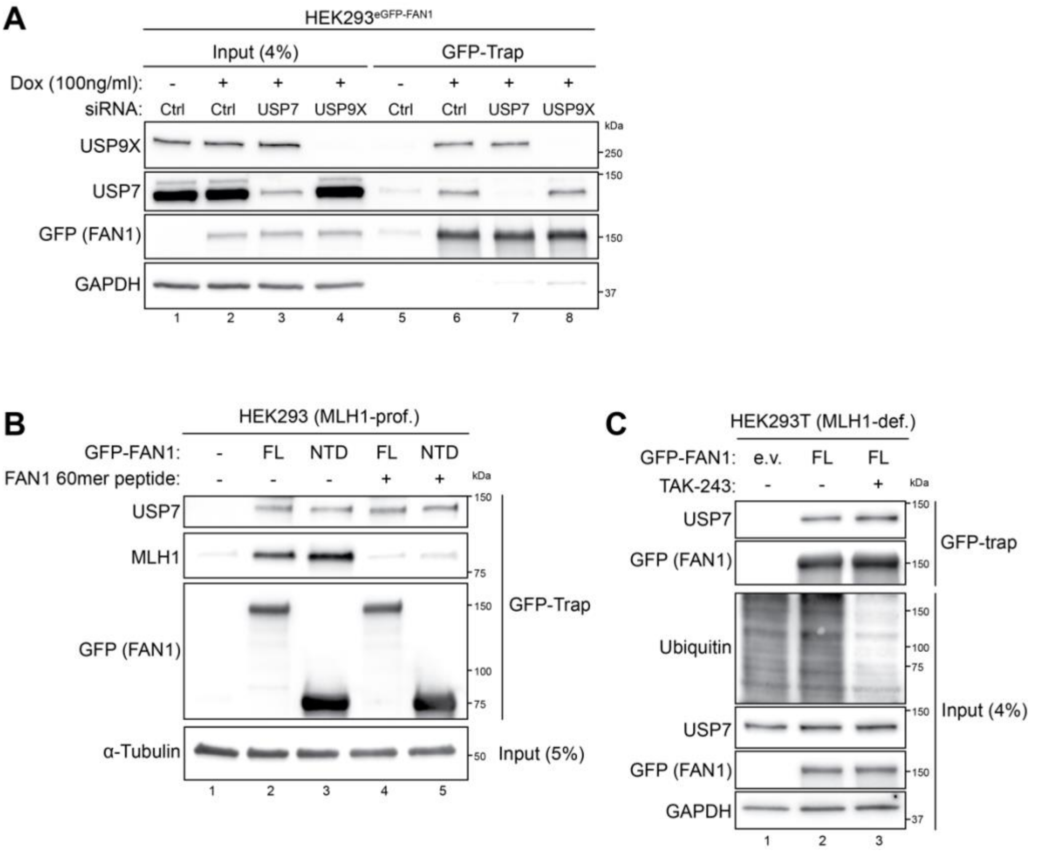

Figure S1 (related to Figure 1). USP7 associate with FAN1 independently of USP9X, MLH1 or ubiquitin. (A) HEK293$^{eGFP\text{-}FAN1}$ cells were transfected either with non-targeting (Ctrl) or the indicated siRNA oligos. 24 h later, GFP-FAN1 expression was induced with Dox (100 ng/ml). 24 h post induction, cells were lysed and whole-cell extracts were subjected to GFP-Trap resin. Inputs and recovered protein complexes were analysed by immunoblotting. (**B**) HEK293 cells were transfected with either e.v. or GFP-FAN1-wt expressing constructs. 48 h later, cells were lysed and whole-cell extracts were incubated with a FAN1-derived 60-mer peptide (aa 118 to 177) for 2 h and subjected to GFP-Trap resin. Inputs and recovered protein complexes were analysed by immunoblotting. (**C**) HEK293T cells were transfected with either e.v. or GFP-FAN1-wt expressing constructs. 48 h later, cells were either mock-treated (-) or treated (+) with TAK-243 (10 μM) for 2 h, lysed and whole-cell extracts were subjected to GFP-Trap resin. Inputs and recovered protein complexes were analysed by immunoblotting. The experiments in (**A**-**C**) were repeated three independent times.

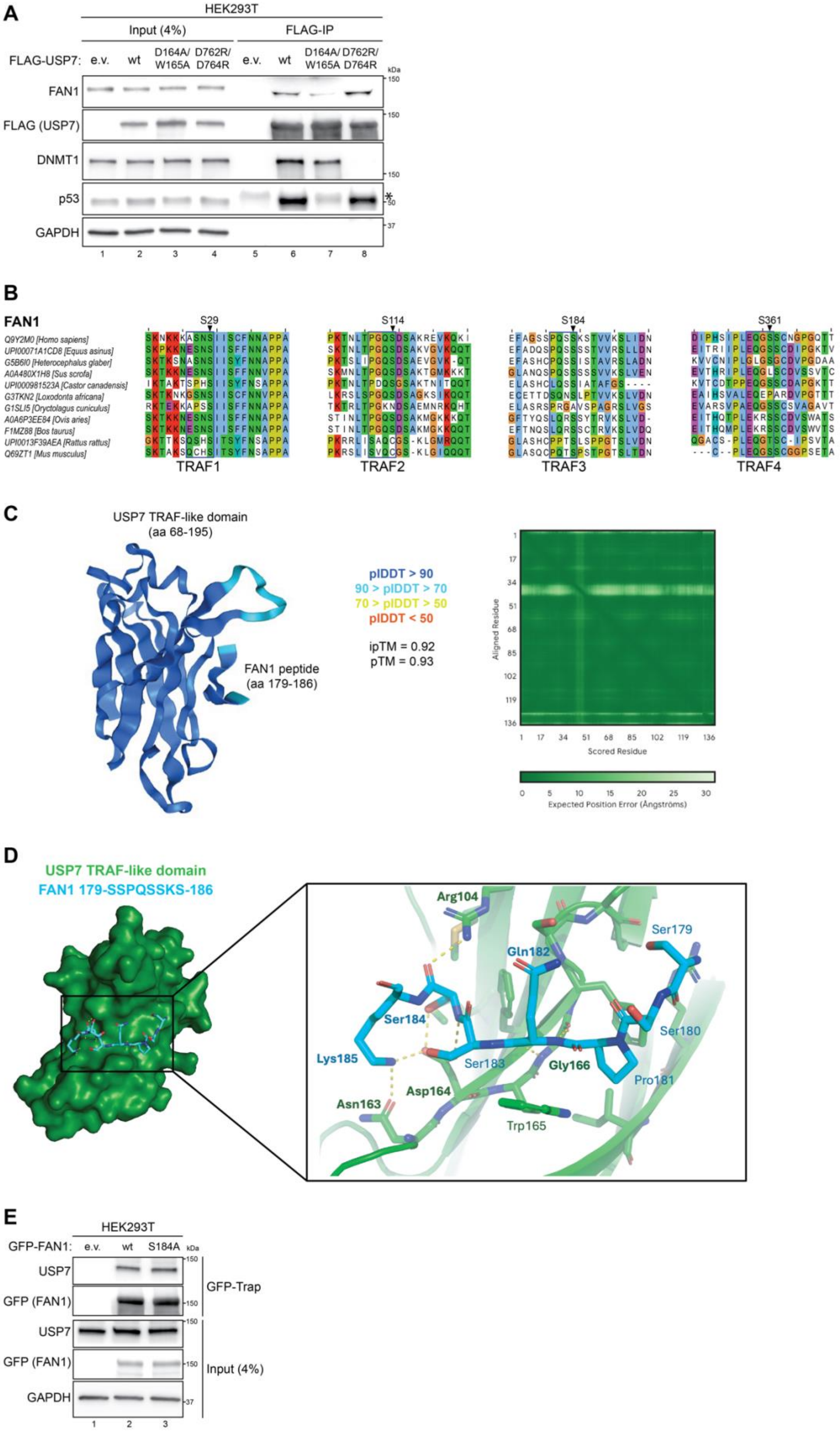
A
HEK293T
Input (4%)
FLAG-IP
FLAG-USP7: e.v. wt D164A/W165A D762R/D764R e.v. wt D164A/W165A D762R/D764R
kDa
FAN1
FLAG (USP7)
DNMT1
p53
GAPDH
150
150
150
*
50
37
1 2 3 4 5 6 7 8
B
FAN1
S29
S114
S184
S361
Q9Y2M0 [Homo sapiens]
UPI00071A1CD8 [Equus asinus]
G5B6I0 [Heterocephalus glaber]
A0A480X1H8 [Sus scrofa]
UPI000981523A [Castor canadensis]
G3TKN2 [Loxodonta africana]
G1SLI5 [Oryctolagus cuniculus]
A0A6P3EE84 [Ovis aries]
F1MZ88 [Bos taurus]
UPI0013F39AEA [Rattus rattus]
Q69ZT1 [Mus musculus]
TRAF1
TRAF2
TRAF3
TRAF4
C
USP7 TRAF-like domain
(aa 68-195)
FAN1 peptide
(aa 179-186)
pIDDT > 90
90 > pIDDT > 70
70 > pIDDT > 50
pIDDT < 50
ipTM = 0.92
pTM = 0.93
Aligned Residue
Scored Residue
1 17 34 51 68 85 102 119 136
0 5 10 15 20 25 30
Expected Position Error (Ångströms)
D
USP7 TRAF-like domain
FAN1 179-SSPQSSKS-186
Arg104
Ser179
Gln182
Ser184
Ser180
Lys185
Ser183
Gly166
Pro181
Asn163
Asp164
Trp165
E
HEK293T
GFP-FAN1: e.v. wt S184A
kDa
USP7
GFP (FAN1)
GFP-Trap
USP7
GFP (FAN1)
GAPDH
Input (4%)
150
150
150
150
37
1 2 3

Figure S2 (related to Figure 2). USP7 contacts FAN1 via its TRAF-like domain. (**A**) HEK293T cells were transfected with either e.v. or the indicated FLAG-USP7 expression constructs. 48 h later, cells were lysed and whole-cell extracts were subjected to IP using anti-FLAG M2 affinity resin. Inputs and recovered protein complexes were analysed by immunoblotting. Asterisk indicates heavy chain IgGs from anti-FLAG M2 affinity resin. (**B**) Selected fragments from a multiple sequence alignment of human FAN1, focused on four serine residues embedded within the P/A/ExxS motif preferentially recognized by USP7 on various partners, are shown using Jalview[81]. Representative mammalian sequences are included, with the blue box highlighting the positions of the conserved motif surrounding serines S29, S114, S184, and S361. (**C**) Schematic representation of the 3D structure of the interaction complex between USP7's TRAF domain (aa 68-195) and FAN1 peptide (aa 179-186) by AlphaFold 3[41], colored according to the predicted local Distance Difference Test (plDDT). Dark blue: regions with pIDDT greater than 90 (Very high); light blue: regions with pIDDT between 70 and 90 (Confident); yellow regions with pIDDT between 50 and 70 (Low); orange: regions with pIDDT lower than 50 (Very low). Interface predicted template modelling (ipTM) = 0.92 and predicted template modelling (pTM) = 0.93. Predicted Aligned Error (PAE) plot indicating regions of high (dark green) and low (light green) confidence. (**D**) Analysis of the interactions mediated in the model predicted in **C** indicate S184, as well as other residues in the FAN1 peptide (cyan), may mediate hydrogen bonds with the TRAF domain of USP7 (green). Residues involved in hydrogen bonds are shown in bold. The experiments in (**A** and **E**) were repeated three independent times.

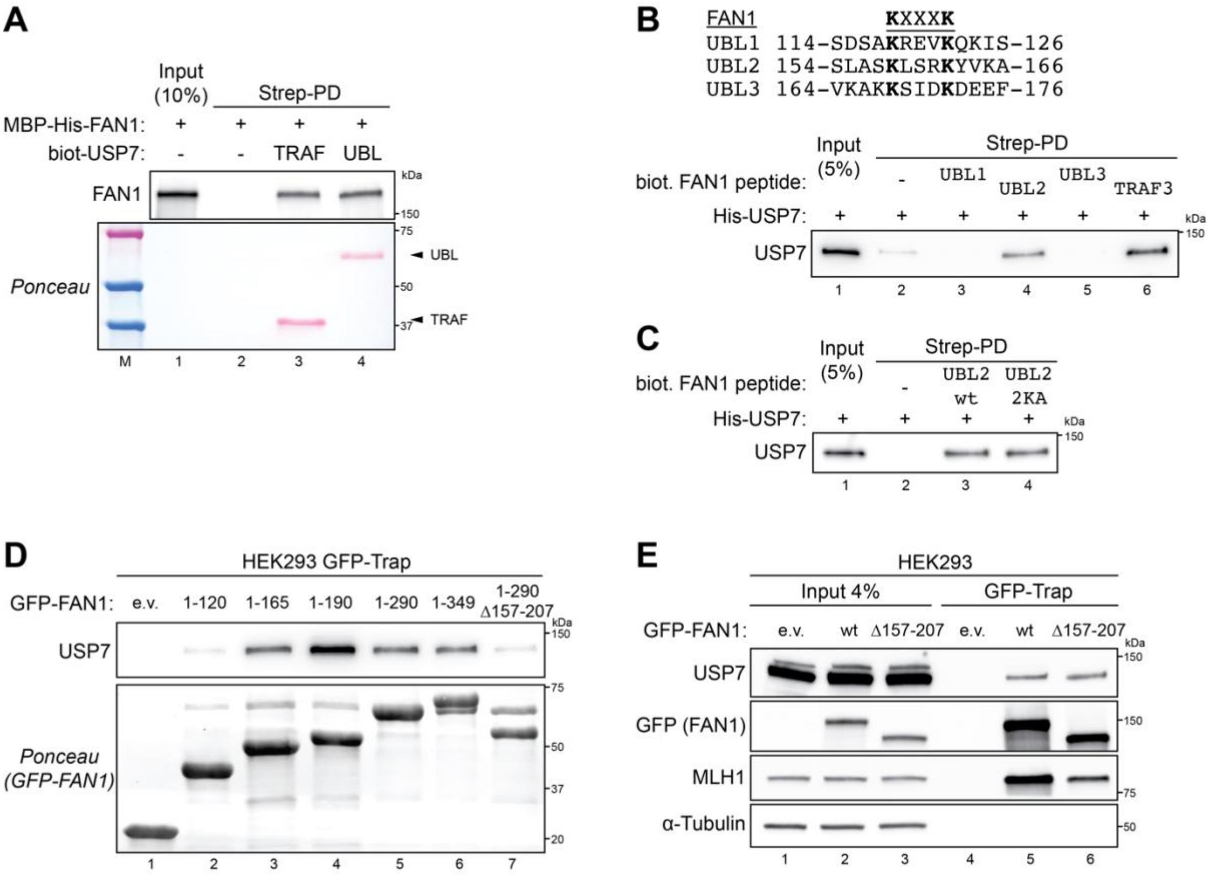

Figure S3 (Related to Figure 2). USP7 contacts FAN1 via its UBL domain. (**A**) HEK293T cells were transfected with either e.v. or the indicated GFP-FAN1 expression constructs. 48 h after transfection, cells were lysed and whole-cell extracts were subjected to GFP-Trap resin. Inputs and recovered protein complexes were analysed by immunoblotting. (**B**) Purified recombinant biotin-tagged USP7 fragments (6 µg) were immobilized on Strep-Tactin®XT-4Flow® resin and incubated with purified recombinant MBP-His-FAN1 (1 µg). Ponceau staining was performed to visualize the USP7 fragments. Inputs and recovered MBP-His-FAN1 were analysed by immunoblotting. (**C**) Sequences of the biotinylated 13-mer FAN1-derived UBL peptides. The corresponding amino acids are indicated. Key residues are highlighted in bold. (**C** and **D**) Biotinylated FAN1 UBL peptides (10 µg) were immobilized on Strep-Tactin® XT 4Flow® resin and incubated with recombinant purified His-USP7. Inputs and recovered His-USP7 were analysed by immunoblotting. (**E**) HEK293 cells were transfected with either e.v. or the indicated GFP-FAN1 expression constructs. 48 h after transfection, cells were lysed and whole-cell extracts were subjected to GFP-Trap resin. Inputs and recovered protein complexes were analysed by immunoblotting. The experiments in (**A**-**E**) were repeated three independent times.

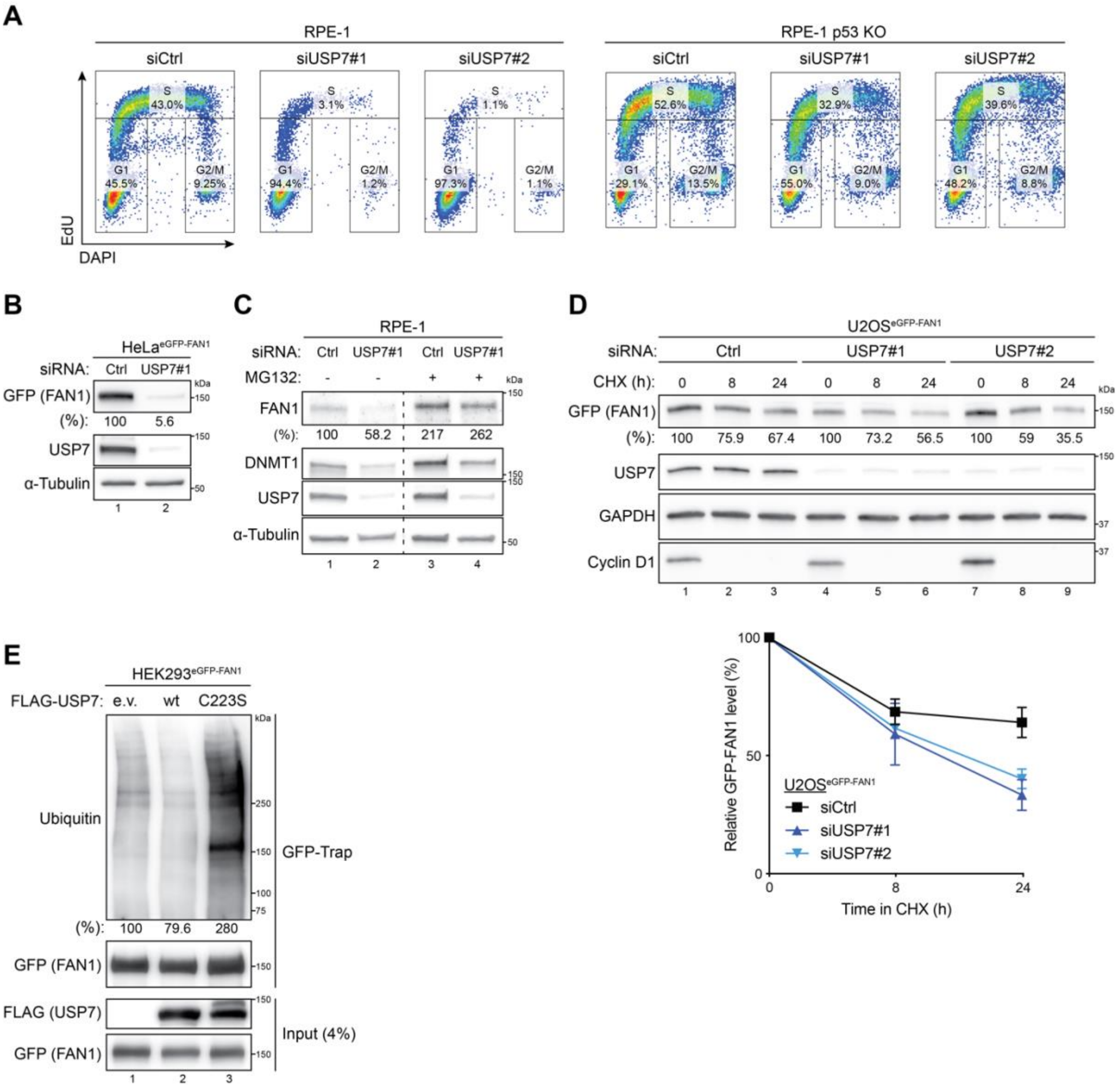

Figure S4 (Related to Figure 3). USP7 controls FAN1 stability via its deubiquitinase activity. (**A**) RPE-1 parental and p53 KO cells were transfected with either non-targeting (Ctrl) or USP7 siRNA oligos. 48 h later EdU was incorporated for 30 min. Cells were harvested, fixed and immunostained with Click-IT reaction and DAPI. Fluorescent intensities were measured by flow cytometry. Cells were gated in FSC-A/SSC-A to discriminate cells from debris, then gated in FSC-H/FSC-A to discriminate single cells. The resulting population was analysed on VL1-A (DAPI-A)/RL1-H (EdU-H) to display cell cycle distribution. Dot plots illustrate the EdU signal intensity (y-axis) versus the DAPI content (x-axis). Gates depict the percentage of cells in G1, S and G2/M. (**B**) Doxycycline-inducible HeLa$^{eGFP\text{-}FAN1}$ cells were transfected with either non-targeting (Ctrl) or USP7 siRNA oligos. 24 h later, GFP-FAN1 expression was induced with Dox (100 ng/ml) for 24 h and whole-cell lysates were analysed by immunoblotting. Relative GFP-FAN1 protein levels were determined by quantification of GFP-FAN1 band intensity (normalized to α-Tubulin) with the ImageJ software. (**C**) RPE-1 parental cells were transfected with either non-targeting (Ctrl) or USP7 siRNA oligos. 48 h later, cells were either mock-treated (-) or treated (+) with MG-132 (10 µM) for 6 h and whole-cell lysates were analysed by immunoblotting. Lanes 3 and 4 were rearranged, as they were not adjacent to lane 1 and 2 in the original blot. Relative FAN1 protein levels

were determined by quantification of FAN1 band intensity (normalized to α-Tubulin) with the ImageJ software. (**D**) Doxycycline-inducible U2OS$^{eGFP-FAN1}$ cells were transfected with either non-targeting (Ctrl) or USP7 siRNA oligos. 24 h later, GFP-FAN1 expression was induced with Dox (100 ng/ml). 24 h post induction, cells were either mock-treated or treated with CHX (100 µg/ml) for the indicated time points and whole-cell lysates were analysed by immunoblotting (top panel). Relative GFP-FAN1 protein levels were determined by quantification of GFP-FAN1 band intensity (normalized to GAPDH) with the ImageJ software (bottom panel; siCtrl: black square; siUSP7#1: blue triangle; siUSP7#2: cyan inverted triangle). Data are presented as the means ± SD of three biological replicates (n=3). (**E**) Doxycycline-inducible HEK293$^{eGFP-FAN1}$ cells were transfected with either e.v. or indicated FLAG-USP7 expression constructs. 24 h later, GFP-FAN1 expression was induced with Dox (100ng/ml). 24 h post induction, cells were lysed and whole-cell extracts were subjected to GFP-Trap resin. Inputs and recovered protein complexes were analysed by immunoblotting. In (**B-D**), relative FAN1 protein levels were determined by quantification of FAN1 band intensity (normalized to α-Tubulin or GAPDH loading controls) with the ImageJ software. In (E), relative ubiquitin levels in the GFP-Trap were determined by quantification of ubiquitin signal intensity (normalized to GFP-FAN1) with the ImageJ software. The experiments in (**B**, **C** and **E**) were repeated three independent times.

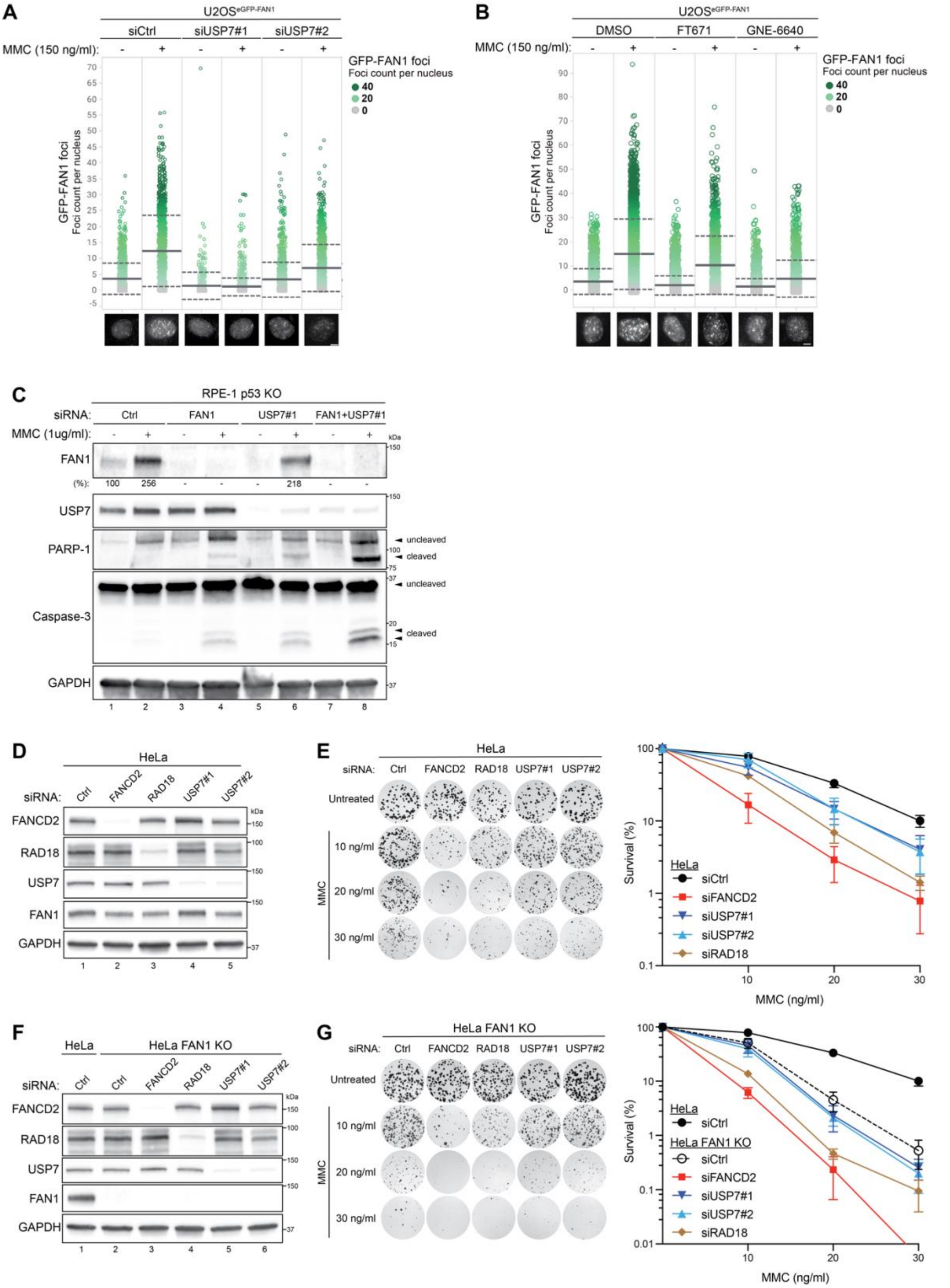

Figure S5 (Related to Figure 4). USP7 facilitates FAN1 localization and function during ICL repair. (**A**) QIBC analysis of chromatin-bound GFP-FAN1 foci in U2OS$^{GFP-FAN1}$ cells transfected with either non-targeting (Ctrl) or USP7 siRNA oligos. 24h later, cells were either mock-treated (-) or treated (+) with MMC (150 ng/ml) for 24 h. Color-coded scatterplots (from grey to green) indicate the number of GFP-FAN1 foci per nucleus from one biological replicate. Mean (solid line) and standard deviation (SD) from

the mean (dashed lines) are indicated. Representative images are shown (bottom). Scale bar: 10 µm. This experiment was repeated three times. (**B**) QIBC analysis of chromatin-bound GFP-FAN1 foci in U2OS$^{GFP\text{-}FAN1}$ cells were concurrently mock-treated (-) or treated (+) with MMC (150 ng/ml) and either FT671 (10 µM) or GNE-6640 (10 µM) for 24 h. Color-coded scatterplots (from grey to green) indicate the number of GFP-FAN1 foci per nucleus from one biological replicate. Mean (solid line) and standard deviation (SD) from the mean (dashed lines) are indicated. Representative images are shown (bottom). Scale bar: 10 µm. This experiment was repeated three times. (**C**) RPE-1 p53 KO cells were transfected either non-targeting (Ctrl) or USP7 siRNA oligos. 24 h later, cells were either mock-treated (-) or treated (+) with MMC (1 µg/ml) for 48 h and whole-cell lysates were analysed by immunoblotting. Arrows indicate respective uncleaved and cleaved forms of PARP-1 and Caspase-3. Relative FAN1 protein levels were determined by quantification of FAN1 band intensity (normalized to GAPDH) with the ImageJ software. The experiment was repeated three independent times. (**D**) Western blot analysis of HeLa cells depleted of the indicated factors. (**E**) Clonogenic survival assay of the HeLa cells depleted of the indicated factors (Ctrl: black circle; FANCD2: red square; USP7#1: inverted blue triangle; USP7#2: cyan triangle; RAD18: brown diamond) and exposed to increasing doses of MMC for 24 h. Viability of untreated cells was defined as 100%. Data are presented as the means ± SD of four biological replicates (n=4). Representative images are shown (left). (**F**) Western blot analysis of HeLa and HeLa FAN1 KO cells depleted of the indicated factors. (**G**) Clonogenic survival assay of HeLa and HeLa FAN1 KO cells depleted of the indicated factors(HeLa, Ctrl: black circle; HeLa FAN1 KO, Ctrl: empty black circle; FANCD2: red square; USP7#1: inverted blue triangle; USP7#2: cyan triangle; RAD18: brown diamond) and exposed to increasing doses of MMC for 24 h. Viability of untreated cells was defined as 100%. Data are presented as the means ± SD of three biological replicates (n=3). Representative images are shown (left).

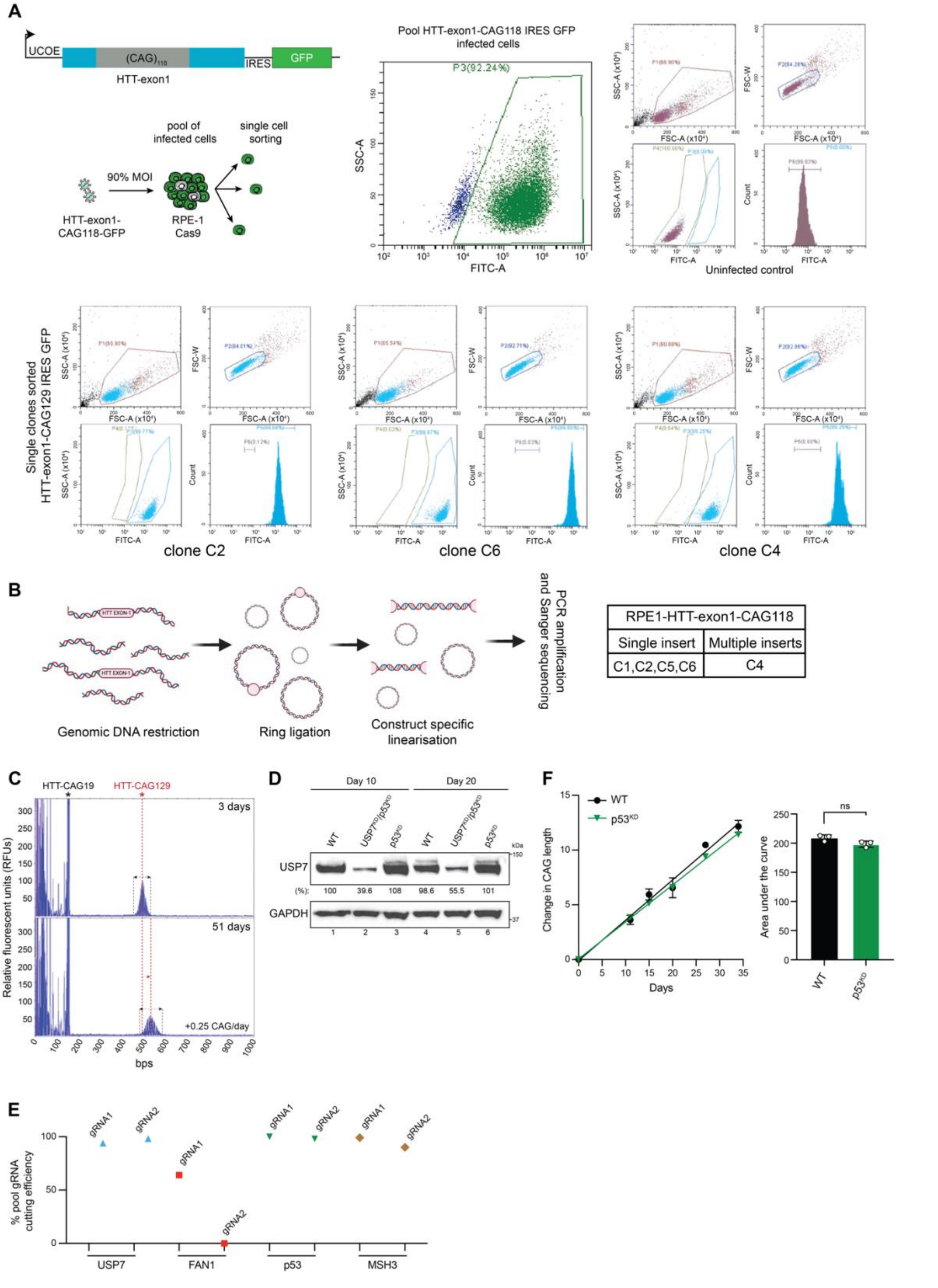

Figure S6 (Related to Figure 5). The RPE-1 HTT-exon1-CAG129 cell line is a rapid-expansion human model of CAG repeat instability. (**A**) Workflow for generating the RPE-1^{CAG129} cell line. *Top:* Schematic of the HTT-exon1-CAG118-IRES-GFP construct containing 118 CAG repeats. RPE-1 Cas9 cells were infected at 90% MOI and single-cell sorted based on GFP fluorescence. FACS plots show GFP intensity

in pooled infected cells versus uninfected controls, and in representative clones (C2, C6, C4). By the time of clone selection, sizing and banking, the CAG expanded to 129 repeats. (**B**) Ring ligation strategy to determine genomic integration. Genomic DNA was digested with restriction enzymes, circularized, re-linearized at construct-specific sites, and analysed by PCR and Sanger sequencing. Clones C1, C2, C5, and C6 contained single integrations; clone C4 carried multiple insertions. Created in BioRender (https://biorender.com/ra2regc). (**C**) Representative fragment analysis traces showing CAG repeat expansion over time in RPE-1^{CAG129} clone C2. Traces from day 3 and day 51 demonstrate a shift in the HTT-CAG129 peak position, indicating progressive repeat expansion. Peaks correspond to the endogenous HTT-CAG19 allele (black asterisk) and the integrated HTT-CAG129 construct (red asterisk). The expansion rate was estimated at +0.25 CAG repeats per day. Red arrow indicates the broadening of the inserted allele peak over time, reflecting heterogeneity in repeat length, a hallmark of ongoing somatic expansion. (**D**) Western blot validation of USP7 knock-down in RPE-1^{CAG129} cells. Whole cell lysates from wt, USP7 KD, p53 KD, and USP7 KD/p53 KD cells were collected at days 10 and 20 post-infection and analysed by immunoblotting. Relative USP7 protein levels were determined by quantification of USP7 band intensity (normalized to GAPDH) with the ImageJ software. (**E**) Quantification of sgRNA cutting efficiency in pooled RPE-1^{CAG129} cells following lentiviral-mediated knock-down of USP7, FAN1, p53 and MSH3, as used in experiments shown in Figure 5. (**F**) CAG repeat expansion over time in RPE-1^{CAG129} cells following *p53* knock-down. Left: Time-course analysis of CAG repeat length in *p53*^{KD} (green) and wt (black) cells. Right: AUC quantification shows no significant difference (ns); bars represent mean ± SD (n=3).

Table S1. List of gRNAs used in this study.

Gene	gRNA name	Source	5'-3' sequence	Cell line used
FAN1	FAN1-466	This study	CACCACAAGGAAACTCCGA	HeLa
FAN1	FAN1-470	This study	AGAAGTGGTTGAAAAACGTG	HeLa
FAN1	FAN1 gRNA1	This study	ATTGTCCAGAAAATACGTAA	RPE-1-Cas9-CAG118
FAN1	FAN1 gRNA2	This study	AATGCAGGCTTTCTACAGAC	RPE-1-Cas9-CAG118
MSH3	MSH3 gRNA1	This study	AAATCCACCTCCTCCTCCACAGG	RPE-1-Cas9-CAG118
MSH3	MSH3 gRNA2	This study	CAGAAGAAAGAAGAGACCATTGG	RPE-1-Cas9-CAG118
TP53	TP53 gRNA1	This study	TCTCGAAGCGCTCACGCCCACGG	RPE-1-Cas9-CAG118
TP53	TP53 gRNA2	This study	CCTTGAGTTCCAAGGCCTCATTC	RPE-1-Cas9-CAG118
USP7	USP7 gRNA1	This study	CCACATAATCCACAAATGATTCA	RPE-1-Cas9-CAG118
USP7	USP7 gRNA2	This study	CCGACGTAGCCTGTGTGCTTCTT	RPE-1-Cas9-CAG118

Table S2. List of DNA primers used in this study.

Primer/oligonucleotide	Source	5'-3' sequence
FAN1_S184A_F	Microsynth	ACAGAGTGCCAAATCCACAGTTGTTAAGAGCC
FAN1_S184A_R	Microsynth	GATTTGGCACTCTGTGGACTAGAACCGG
FAN1_1-372_F	Microsynth	CAACCGGTTAACTCGAGTCTAGAGGGCCC
FAN1_1-372_R	Microsynth	CGAGTTAACCGGTTGTTTGACCAGG
FAN1_del1-372_F	Microsynth	GCCGCATGCCTTACTACCTTCGGAGTTTCCTTG
FAN1_del1-372_R	Microsynth	AGTAAGGCATGCGGCCGCTGTACTTG
FAN1_del1-40_F	Microsynth	GCCGCATGAAACTTGCCTGCCCCGTTTG
FAN1_del1-40_R	Microsynth	CAAGTTTCATGCGGCCGCTGTACTTG
FAN1_del157-207_F	Microsynth	GCCTAGCAAACAGTTCTCAAAAAGAAAACGTG
FAN1_del157-207_R	Microsynth	AACTGTTTGCTAGGCTTCCCAAACAAATG
USP7_TRAF_F	Microsynth	TTGCGTGGTGAGGATCCGGAATTCCGCC
USP7_TRAF_R	Microsynth	ATCCTCACCACGCAACTCCATGGGG
USP7_delUBL_F	Microsynth	GGCAGGAATGAGGATCCGGAATTCCGCC
USP7_delUBL_R	Microsynth	ATCCTCATTCCTGCCGCTCCTTCCG
USP7_UBL_F	Microsynth	CCGGTATGGATATTCCTCAGCAGTTGGTGGAG
USP7_UBL_R	Microsynth	GAATATCCATACCGGTCTTGTCATCGTC
USP7_D164A_W165A_F	Microsynth	AAAATGCGGCGGGATTTTCCAATTTTATGGCCTGG
USP7_D164A_W165A_R	Microsynth	ATCCCGCCGCATTTTCTTTATGGAAGAACAAATGAC
USP7_C223S_F	Microsynth	AGCGACTAGCTACATGAACAGCCTGCTACAGACG
USP7_C223S_R	Microsynth	ATGTAGCTAGTCGCTCCCTGATTCTTTAAGCC

HD3 FAM	Merck, Teo *et al.*, 2008	CCTTCGAGTCCCTCAAGTCCTT
HD5	Merck, Mangiarini *et al.*, 1996	CGGCTGAGGCAGCAGCGGCTGT

Table S3. List of siRNA oligonucleotides used in this study.

Oligonucleotide	Sequence	Source	Concentration used
siCrtl	CGUACGCGGAAUACUUCGA	Microsynth	40 nM
siFAN1	GUAAGGCUCUUUCAACGUA	Microsynth<br>MacKay *et al.,* 2010	40 nM
siFANCD2	CAGAGUUUGCUUCACUCUCUA	Microsynth<br>Enoiu *et al.,* 2012	40 nM
siRad18	AUGGUUGUUGCCCGAGGUU	Microsynth<br>Porro *et al.,* 2017	40 nM
siUSP7#1	GUGUAAAGAAGUAGACUAU	Microsynth<br>Jiang *et al.,* 2017	40 nM
siUSP7#2	CCCAAAUUAUUCCGCGGCAAA	Microsynth<br>Zlatanou *et al.,* 2016	40 nM
siUSP9X	CUGUGAUUCAGCAACUCUA	Microsynth<br>Shang *et al.,* 2019	40 nM
siUSP11	AAGCGUUACUAUGACGAGGUAUU	Microsynth<br>Wiltshire *et al.,* 2010	40 nM
siUSP48	GCGUAAGCAAAGUGUGGAUA	Microsynth<br>Uckelmann *et al.,* 2018	40 nM

Table S4. List of antibodies used in this study.

Antibody	Species	Source	Application	Dilution
Caspase-3	Rabbit	Cell Signaling (9662)	IB	1:1000
Cyclin D1	Rabbit	Cell Signaling (2922)	IB	1:1000
DNMT1	Rabbit	Cell Signaling (5032)	IB	1:1000
FAN1	Rabbit	ProteinTech (17600-1-AP)	IB	1:1000
FAN1	Mouse	Genscript (1A11-2-A)	IB and *PLA*	1:500<br>*1:100*
FAN1	Rabbit	Novus (NBP1-42677)	IP	1:1000
FAN1	Goat	MacKay et al. (2010)	IF	1:200
FANCD2	Rabbit	Novus (NB-100-182)	IB and *IF*	1:5000<br>*1:250*
FLAG M2	Mouse	Sigma-Aldrich (#F1804)	IB	1:1000
GAPDH	Mouse	Millipore (MAB374)	IB	1:40000
GFP	Rabbit	Abcam (ab290)	IB	1:1000

GFP	Mouse	Roche (11814460001)	IF	1:200
GST	Rabbit	Abcam (ab9085)	IB	1:500
HA	Mouse	Santa Cruz (sc-7392)	IB	1:1000
Lamin B1	Rabbit	Abcam (#ab16048)	IB	1:1000
MLH1	Rabbit	Abcam (ab92312)	IB	1:5000
p53	Mouse	Santa Cruz (sc-126)	IB	1:4000
PARP-1	Rabbit	Abcam (ab227244)	IB	1:1000
RAD18	Rabbit	Cell Signaling (9040)	IB	1:1000
RNF169	Rabbit	Aviva Systems Biology Corp (ARP43508_P050)	IB	1:1000
Ubiquitin	Mouse	Santa Cruz (sc-8017)	IB	1:1000
USP7	Rabbit	Bethyl (A300-033A)	IB	1:10000
USP9X	Rabbit	Bethyl (A301-351A-T)	IB	1:2000
USP11	Rabbit	Bethyl (A301-613A)	IB	1:2000
USP48	Rabbit	Abcam (ab72226)	IB	1:2000
α-Tubulin	Mouse	Sigma-Aldrich (#T9026)	IB	1:20000

Table S5. List of peptides used in this study.

**Peptide**	**Sequence**	**Source**
TRAF1 (12mer)	biotin-NKKKASNSIISV-NH2	This study (SynPeptide)
TRAF2 (12mer)	biotin-TNLTPGQSDSAK-NH2	This study (SynPeptide)
TRAF3 (12mer)	biotin-AGSSPQSSKSTV-NH2	This study (SynPeptide)
TRAF3 S184A (12mer)	biotin-AGSSPQSAKSTV-NH2	This study (SynPeptide)
TRAF3 (8mer)	FITC-Ahx-SSPQSSKS	This study (GenScript)
TRAF3 P181A (8mer)	FITC-Ahx-SSAQSSKS	This study (GenScript)
TRAF3 Q182A (8mer)	FITC-Ahx-SSPASSKS	This study (GenScript)
TRAF3 S184A (8mer)	FITC-Ahx-SSPQSAKS	This study (GenScript)
TRAF4 (12mer)	biotin-PLEQGSSCNGPG-NH2	This study (SynPeptide)
UBL1 (12mer)	biotin-SDSAKREVKQKIS-NH2	This study (SynPeptide)
UBL2 (12mer)	biotin-SLASKLSRKYVKA-NH2	This study (SynPeptide)
UBL2 2KA (12mer)	biotin-SLASALSRAYVKA-NH2	This study (SynPeptide)
UBL3 (12mer)	biotin-VKAKKSIDKDEEF-NH2	This study (SynPeptide)
FAN1 60mer	human FAN1 (aa 118-177)	Porro *et al.*, 2021 (GenScript)
