# Supplementary material for: USP7 deubiquitinase stabilizes FAN1 to support DNA crosslink repair and suppress CAG repeat expansion
Source: Nat Commun. 2026 Mar 6;17:3551. doi: 10.1038/s41467-026-70051-9 (PMC13086964; doi:10.1038/s41467-026-70051-9)
Supplement: Supplementary file 2 — Description of Additional Supplementary Files [file 41467_2026_70051_MOESM2_ESM.docx]

**Description of Additional Supplementary Files**

**Supplementary Data 1:** Proteins identified to interact with FAN1 by mass spectrometry in HEK293 cells
