# Supplementary material for: USP7 deubiquitinase stabilizes FAN1 to support DNA crosslink repair and suppress CAG repeat expansion
Source: Nat Commun. 2026 Mar 6;17:3551. doi: 10.1038/s41467-026-70051-9 (PMC13086964; doi:10.1038/s41467-026-70051-9)
Supplement: Supplementary file 4 — Reporting Summary [file 41467_2026_70051_MOESM4_ESM.pdf]

nature portfolio

Corresponding author(s): Gabriel Balmus, Alessandro A Sartori
Last updated by author(s): Jan 23, 2026

Reporting Summary

Nature Portfolio wishes to improve the reproducibility of the work that we publish. This form provides structure for consistency and transparency in reporting. For further information on Nature Portfolio policies, see our Editorial Policies and the Editorial Policy Checklist.

Statistics

For all statistical analyses, confirm that the following items are present in the figure legend, table legend, main text, or Methods section.

n/a	Confirmed	
☐	☒	The exact sample size (*n*) for each experimental group/condition, given as a discrete number and unit of measurement
☐	☒	A statement on whether measurements were taken from distinct samples or whether the same sample was measured repeatedly
☐	☒	The statistical test(s) used AND whether they are one- or two-sided<br>*Only common tests should be described solely by name; describe more complex techniques in the Methods section.*
☒	☐	A description of all covariates tested
☒	☐	A description of any assumptions or corrections, such as tests of normality and adjustment for multiple comparisons
☐	☒	A full description of the statistical parameters including central tendency (e.g. means) or other basic estimates (e.g. regression coefficient) AND variation (e.g. standard deviation) or associated estimates of uncertainty (e.g. confidence intervals)
☐	☒	For null hypothesis testing, the test statistic (e.g. *F*, *t*, *r*) with confidence intervals, effect sizes, degrees of freedom and *P* value noted<br>*Give P values as exact values whenever suitable.*
☒	☐	For Bayesian analysis, information on the choice of priors and Markov chain Monte Carlo settings
☒	☐	For hierarchical and complex designs, identification of the appropriate level for tests and full reporting of outcomes
☒	☐	Estimates of effect sizes (e.g. Cohen's *d*, Pearson's *r*), indicating how they were calculated

Our web collection on statistics for biologists contains articles on many of the points above.

Software and code

Policy information about availability of computer code

Data collection

High-content microscopy and fluorescence microscopy: Olympus ScanR Image Acquisition software (versions 3.2 and 3.3.0); Leica Application Suite X (version 3.10.1.29575).
Immunoblotting: FusionCapt Advance Solo 7
Colony formation assay: AlphaImager HP (version 3.4.0)
Flow cytometry: Attune NxT Software (version 4.2.0)
Fluorescence polarization: Agilent BioTek Gen5 (versions 3.03.14 and 3.16.10)
GeneMapper® Software v6.0 (Applied Biosystems)

Data analysis

Microscopy image analysis software: Olympus ScanR Analysis (versions 3.2 and 3.3.0), ImageJ2 (Fiji) (version 2.16.0/1.54n, 1.54p); Cell Profiler (version 4.2.6).
Microscopy data visualization: TIBCO Spotfire (version 10.10.1); GraphPad Prism 10 (version 10.2.0, in situ Proximity ligation assay)
Colony formation assay software: ImageJ Plugin ColonyArea (Guzman et al., Plos One 2014 19 Mar, 9(3):e92444;) GraphPad Prism 10 (version 10.2.0)
Immunoblotting: Adobe Photoshop 21.2.24; ImageJ2 (Fiji) (version 2.16.0/1.54n, 1.54p); GraphPad Prism 10 (version 10.2.0)
Flow cytometry: FlowJo (version 10.10.0)
Proteomics: Fragpipe v17, R using the package prolfqua and Excel v16.88 for data representation.
Fluorescence polarization: GraphPad Prism 10 (version 10.2.0)
CAG repeat expansion: GraphPad Prism 10 (version 10.2.0)
Protein sequence alignment: MMseqs2 (version edb8223d1ea07385ffe63d4f103af0eb12b2058e, from https://github.com/soedinglab/

MMseqs2), hhfilter (version 3.3.0, from https://github.com/soedinglab/hh-suite), MAFFT (version 7.475, from https://mafft.cbrc.jp/alignment/software/), Jalview (version 2.11.4.1 git-commit:6d1fc3490c, from https://www.jalview.org/)
Protein structure prediction: AlphaFold 3 (https://doi.org/10.1038/s41586-024-07487-w) and PyMOL Molecular Graphics System (version 3.1.4.1)
For CAG repeat expansion, peak height and area data exported from GeneMapper were subjected to statistical and graphical analysis in R v4.3.1 (R Core Team, 2023) using packages tidyverse (v2.0), ggplot2 (v3.4), dplyr (v1.1), and lme4 (v1.1). Data were normalized to the internal control peak or total signal, and run-to-run variation was assessed using size standard reference peak

For manuscripts utilizing custom algorithms or software that are central to the research but not yet described in published literature, software must be made available to editors and reviewers. We strongly encourage code deposition in a community repository (e.g. GitHub). See the Nature Portfolio guidelines for submitting code & software for further information.

Data

Policy information about availability of data

All manuscripts must include a data availability statement. This statement should provide the following information, where applicable:

- Accession codes, unique identifiers, or web links for publicly available datasets
- A description of any restrictions on data availability
- For clinical datasets or third party data, please ensure that the statement adheres to our policy

Raw proteomics data were deposited in the PRIDE database, with the dataset identifier PXD065101.
Raw data used to build all graphs and derive statistics, as well as original and uncropped blots, will be available in the provided Source data file.
Microscopy and colony formation images will be made available upon request.

Research involving human participants, their data, or biological material

Policy information about studies with human participants or human data. See also policy information about sex, gender (identity/presentation), and sexual orientation and race, ethnicity and racism.

Reporting on sex and gender	n/a
Reporting on race, ethnicity, or other socially relevant groupings	n/a
Population characteristics	n/a
Recruitment	n/a
Ethics oversight	n/a

Note that full information on the approval of the study protocol must also be provided in the manuscript.

Field-specific reporting

Please select the one below that is the best fit for your research. If you are not sure, read the appropriate sections before making your selection.

[x] Life sciences [ ] Behavioural & social sciences [ ] Ecological, evolutionary & environmental sciences

For a reference copy of the document with all sections, see nature.com/documents/nr-reporting-summary-flat.pdf

Life sciences study design

All studies must disclose on these points even when the disclosure is negative.

Sample size	Sample size for QIBC experiments were determined based on current standard in the field (e.g. Toledo et al., Cell 2013 Nov 21, 155 (5):1088-1103; Gatti et al., Cell Reports 2020 Aug 4, 32(5):107985; Lezaja et al., Nat. Commun. 2021 Jun 22, 12(1):3827).<br>QIBC experiments: >1000 cells per condition.<br>PLA experiments: >250 cells per condition.<br>Sample size (or number of repeats) for any other experiment was chosen based on what is common in the field.
Data exclusions	No relevant data were excluded from this study.
Replication	Experiments were performed and repeated in at least 2 to 3 biological replicates. Reproduced trends of representative data are shown in the figures.
Randomization	Experiments were conducted in asynchronous cycling cell, controls and experimental groups were assigned randomly. For high-content microscopy non-overlapping fields of view were applied for automated sample acquisitions.
Blinding	Data collection and data analysis of microscopy experiments were conducted using automated image acquisition.

Reporting for specific materials, systems and methods

We require information from authors about some types of materials, experimental systems and methods used in many studies. Here, indicate whether each material, system or method listed is relevant to your study. If you are not sure if a list item applies to your research, read the appropriate section before selecting a response.

Materials & experimental systems

n/a	Involved in the study
☐	☒ Antibodies
☐	☒ Eukaryotic cell lines
☒	☐ Palaeontology and archaeology
☒	☐ Animals and other organisms
☒	☐ Clinical data
☒	☐ Dual use research of concern
☒	☐ Plants

Methods

n/a	Involved in the study
☒	☐ ChIP-seq
☐	☒ Flow cytometry
☒	☐ MRI-based neuroimaging

Antibodies

Antibodies used

Primary antibodies used in this study:
Caspase-3 (Cell Signaling, 9662, IB 1:1000)
Cyclin D1 (Cell Signaling, 2922, IB 1:1000)
DNMT1 (Cell Signaling, 5032, IB 1:1000)
FAN1 (ProteinTech, 17600-1-AP, IB 1:1000)
FAN1 (Genscript, 1A11-2-A, IB 1:500 and PLA 1:100)
FAN1 (Novus, NBP1-42677, IP 1:1000)
FAN1 (MacKay et al. (2010) IF 1:200)
FANCD2 (Novus, NB-100-182, IB 1:5000 and IF 1:250)
FLAG M2 (Sigma-Aldrich, #F1804, IB 1:1000)
GAPDH (Millipore, MAB374, IB 1:40000)
GFP (Abcam, ab290, IB 1:1000)
GFP (Roche, 11814460001, IF 1:200)
GST (Abcam, ab9085, IB 1:500)
HA (Santa Cruz, sc-7392, IB 1:1000)
Lamin B1 (Abcam, #ab16048, IB 1:1000)
MLH1 (Abcam, ab92312, IB 1:5000)
p53 (Santa Cruz, sc-126, IB 1:4000)
PARP-1 (Abcam, ab227244, IB 1:1000)
RAD18 (Cell Signaling, 9040, IB 1:1000)
RNF169 (Aviva Systems Biology Corp, ARP43508_P050, IB 1:1000)
Ubiquitin (Santa Cruz, sc-8017, IB 1:1000)
USP7 (Bethyl, A300-033A, IB 1:10000)
USP9X (Bethyl, A301-351A-T, IB 1:2000)
USP11 (Bethyl, A301-613A, IB 1:2000)
USP48 (Abcam, ab72226, IB 1:2000)
α-Tubulin (Sigma-Aldrich, #T9026, IB 1:2000)

Secondary antibodies used in this study:
Alexa Fluor 488 Goat Anti-Rabbit (Thermo Fisher Scientific, A11034, IF 1:500)
Alexa Fluor 647 Goat Anti-Rabbit (Thermo Fisher Scientific, A21244 IF 1:500)
Duolink® In Situ PLA® Probe Anti-Mouse PLUS (Sigma-Aldrich, DUO92001, 1X for PLA)
Duolink® In Situ PLA® Probe Anti-Rabbit MINUS (Sigma-Aldrich, DUO92005, 1X for PLA)
Rabbit IgG HRP Linked Whole Ab (Cytiva, NA934, IB 1:5000)
Mouse IgG HRP Linked Whole Ab (Cytiva, NA931, IB 1:5000)

Validation

The Caspase-3 antibody was cited 5473 and previously validated using KO cell lines as well as etoposide, staurosporine or cytochrome C treatments as stated by the manufacturer: https://www.cellsignal.com/products/9662/datasheet?images=1&protocol=0&size=A4

The Cyclin D1 antibody was cited 686 times as reported in the manufaturer's website: https://www.cellsignal.com/products/primary-antibodies/cyclin-d1-antibody/2922?srsltid=AfmBOopAzz7fex881BbdbreD7y2teEG3wqSIjFSQ5cLi4zTYzJn9kddt

The DNMT1 antibody was cited 226 times as reported in the manufaturer's website: https://www.cellsignal.com/products/primary-antibodies/dnmt1-d63a6-xp-rabbit-mab/5032?srsltid=AfmBOopd2Vk2ju276r_NlADolm0cqCFpcDMgGmOK1JYsWypesQ-2W-B5

All FAN1 antibodies were tested in house for Western Blotting and immunofluorescence using siRNAs and KO cell lines.

The FANCD2 antibody was validated in house using siRNAs and MMC treatment by Western Blotting and immunofluorescence, as well as previously validated using siRNAs as well as AICAR and treatments as stated by the manufacturer: https://www.novusbio.com/products/fancd2-antibody_nb100-182

The FLAG M2 antibody was cited 9676 times as stated on the manufacturer's website: https://www.sigmaaldrich.com/CH/en/product/sigma/f1804?srsltid=AfmBOopLeFMgvSsQqqU4nRV9HmLw-LwxgHh2IAv8y0uQHhIYWfjqyGS6

The GAPDH antibody was cited 3155 times as stated on the manufacturer's website: https://www.sigmaaldrich.com/CH/en/product/mm/mab374?srsltid=AfmBOooy6uvOHmVuJMEAW9zYe3mvx-crdTeWYtxPf4alK4qRyFD228W-

The GFP antibody used for immunoblotting was cited 3369 times as stated on the manufacturer's website: https://www.abcam.com/en-us/products/primary-antibodies/gfp-antibody-ab290?srsltid=AfmBOordbzkFkkyq0ErJSRTCizYj1elfo9ZfP_wto7SCYkqVBI_JbOLa

The GFP antibody used for immunofluorescence was tested in house using cell inducible lines expressing different GFP-tagged proteins, as well as being cited 1127 times as stated on the manufacturer's website: https://www.sigmaaldrich.com/CH/en/product/roche/11814460001?srsltid=AfmBOoq_TD8F27uyDxPbsh6p2kmuE99x9-MSemH-JPGcLX1vGZm5TYgH

The GST antibody was tested using several GST-tagged recombinant proteins, as well as being cited 65 times as stated on the manufacturer's website: https://www.abcam.com/en-us/products/primary-antibodies/gst-antibody-ab9085?srsltid=AfmBOopDZ24DvdfVpBjg_jWVvFEAPNhG8FqDlK5Fdueodbgz_vxMod3S

The HA antibody was tested in house for immunoblotting by overexpressing HA-tagged proteins, as well as being cited 3030 times as stated on the manufacturer's website: https://www.scbt.com/p/ha-probe-antibody-f-7?srsltid=AfmBOopEy5rVanHaWPO7IjmE-FIKeDTcEjUHZeVNmd2MSJWvWleqSuyH

the LaminB1 antibody was validated using KO cell lines as well as being cited 1181 times as stated on the manufacturer's website: https://www.abcam.com/en-us/products/primary-antibodies/lamin-b1-antibody-nuclear-envelope-marker-ab16048?srsltid=AfmBOoqx-PnFaMh88_oZqxv6akqDmxa6Id9kvq-7r8G61gXFbtRWYdNO

The MLH1 antibody was validated in house using siRNAs and different MLH1 KO cell lines, as well as being validated by the manufacturer with KO cell lines and being cited 83 times as stated on the manufacturer's website: https://www.abcam.com/en-us/products/primary-antibodies/mlh1-antibody-epr3894-ab92312?srsltid=AfmBOopnn32K0Gb-vFzyxN5dQLwaYBD3dBo2ZqxB9j2cB1rc5cVRszgx

The p53 antibody was validated in house using p53 KO cell lines, as well as being cited 6657 times as stated on the manufaturer's website: https://www.scbt.com/p/p53-antibody-do-1?srsltid=AfmBOoooGJ1JOPt1X8z0uW6damlFVBawsGzfss-7FIZmXYmyPlSkVZZE

The PARP1 antibody was validated in house treating cells with MMC, as well as being cited 45 times as stated in the manufacturer's website: https://www.abcam.com/en-us/products/primary-antibodies/parp1-antibody-ab227244?srsltid=AfmBOoqHV0cpld9t_GlCQM8hbWGej6q8BvRoKFzFWSDEQaxBGz9key-L

The RAD18 antibody was validated in house using siRNAs, as well as being cited 35 times as stated on the manufacturer's website: https://www.cellsignal.com/products/primary-antibodies/rad18-d2b8-xp-rabbit-mab/9040?srsltid=AfmBOopXtIzfQGsxBClViKK1XY-S0nr95xs5zxrGy9OrmS_od78TT0bh

The ubiquitin antibody was validated in house using an E1 inhibitor, as well as being cited 3423 times as stated in the manufacturer's website: https://www.scbt.com/p/ubiquitin-antibody-p4d1?rsltid=AfmBOop6Ro4q9uA_qCuPeudgACnYEHYQaL5FEIfZ4BBmqDtDUH5-EpV1

The USP7 antibody was validated in house using siRNAs and USP7 KO cell lines, as well as overexpression constructs and recombinant proteins.

The USP9X antibody was validated in house using siRNAs.

The USP11 antibody was validated in house using siRNAs and USP11 KO cell lines, as well as overexpression constructs.

The USP48 antibody was validated in house using siRNAs as well as being cited 10 times as stated on the manufacturer's website: https://www.abcam.com/en-us/products/primary-antibodies/usp48-antibody-ab72226?srsltid=AfmBOopB6K_qN6lPWo6txqwa53XEtMmMYW8SoXh6oEAW1zOjwsnxIqLc

The α-Tubulin antibody was cited 4683 times as stated on the manufacturer's website: https://www.sigmaaldrich.com/CH/en/product/sigma/t9026?srsltid=AfmBOoqByVjlk6IpsblWhDJVKur3UY1pNEJXPmfub67Tf1nM9HnNd1RP

The Alexa Fluor 488 Goat Anti-Rabbit antibody was validated by the manufacturer and cited 7892 times as stated on the manufacturer's website: https://www.thermofisher.com/antibody/product/Goat-anti-Rabbit-IgG-H-L-Highly-Cross-Adsorbed-Secondary-Antibody-Polyclonal/A-11034

The Alexa Fluor 647 Goat Anti-Rabbit antibody was validated by the manufacturer and cited 1932 times as stated on the manufacturer's website: https://www.thermofisher.com/antibody/product/Goat-anti-Rabbit-IgG-H-L-Cross-Adsorbed-Secondary-Antibody-Polyclonal/A-21244

The Duolink® In Situ PLA® Probe Anti-Mouse PLUS was validated by the manufacturer and cited 568 times as stated on the manufacturer's website: https://www.sigmaaldrich.com/CH/en/product/sigma/duo92001?srsltid=AfmBOopqtjBk8rrJEt8_rQ5fuzMuQgWGDgE15n_AcipJv0VsvJgaNdB9

The Duolink® In Situ PLA® Probe Anti-Rabbit MINUS was validated by the manufacturer and cited 568 times as stated on the manufacturer's website: https://www.sigmaaldrich.com/CH/en/product/sigma/duo92005?srsltid=AfmBOoq7U3ikj1yQu3H9B-TX1MAhIJnRwdgp-uQPhM4MoYsWhBwcFdji

The Rabbit or Mouse IgG HRP Linked Whole Ab were extensively tested in house against several rabbit or mouse antibodies, respectively.

Eukaryotic cell lines

Policy information about cell lines and Sex and Gender in Research

Cell line source(s)	U2OS, HEK293, HEK293T, HeLa and RPE-1 cells were purchased from American Type Culture Collection (ATCC).<br>U2OS Flp-In T-REx, HEK293 Flp-In T-REx and HeLa Flp-In T-REx were purchased from Invitrogen, Life Technologies.<br>HeLa FAN1 knock-out cells were generated using the CRISPR/Cas9 system as described in the Material and Methods section.<br>USP7, p53, FAN1 and MSH3 knock-down cells were generated using the CRISPR/Cas9 system as described in the Material and Methods section.
Authentication	None of the cell lines were authenticated in house for this manuscript.
Mycoplasma contamination	All our cell lines are subjected to mycoplasma testing in house.
Commonly misidentified lines (See ICLAC register)	The HeLa cell line was the only commonly misidentified line used in this study.

Plants

Seed stocks	*Report on the source of all seed stocks or other plant material used. If applicable, state the seed stock centre and catalogue number. If plant specimens were collected from the field, describe the collection location, date and sampling procedures.*
Novel plant genotypes	*Describe the methods by which all novel plant genotypes were produced. This includes those generated by transgenic approaches, gene editing, chemical/radiation-based mutagenesis and hybridization. For transgenic lines, describe the transformation method, the number of independent lines analyzed and the generation upon which experiments were performed. For gene-edited lines, describe the editor used, the endogenous sequence targeted for editing, the targeting guide RNA sequence (if applicable) and how the editor was applied.*
Authentication	*Describe any authentication procedures for each seed stock used or novel genotype generated. Describe any experiments used to assess the effect of a mutation and, where applicable, how potential secondary effects (e.g. second site T-DNA insertions, mosiacism, off-target gene editing) were examined.*

Flow Cytometry

Plots

Confirm that:

- [x] The axis labels state the marker and fluorochrome used (e.g. CD4-FITC).
- [x] The axis scales are clearly visible. Include numbers along axes only for bottom left plot of group (a 'group' is an analysis of identical markers).
- [x] All plots are contour plots with outliers or pseudocolor plots.
- [x] A numerical value for number of cells or percentage (with statistics) is provided.

Methodology

Sample preparation	For cell cycle analysis: Prior to acquisition, cells were incubated with 10 µM 5-Ethynyl-2'-deoxyuridine (EdU) for 30 min at 37° C. Cells were then harvested by trypsinization, washed, and fixed in 4% Formaldehyde in PBS. EdU labelling was carried out using the Click-iT EdU technology (Thermo Fisher Scientific) as described in the manufacturer's instructions. DNA was stained by incubating the cells in 1 % BSA/PBS containing 0.1 mg/ml RNase and 1 µg/ml DAPI.
Instrument	For cell cycle analysis: Attune NxT flow cytometer
Software	For cell cycle analysis: data was acquired via Attune NxT Software v4.2.0 and analysed via FlowJo v10.10.0
Cell population abundance	For cell cycle analysis: a minimum of 20,000 events were recorded

Gating strategy	For cell cycle analysis: Initial gates - FSC-A/SSC-A to discriminate cells from debris; cells were gated in FSC-H/FSC-A to discriminate single cells; Resulted population were analyzed on VL1-A (DAPI-A)/RL1-H (EdU-H) to display cell cycle distribution.

☐ Tick this box to confirm that a figure exemplifying the gating strategy is provided in the Supplementary Information.
